# Challenges in the Setup of Large-scale Next-Generation Sequencing Analysis Workflows

**DOI:** 10.1016/j.csbj.2017.10.001

**Published:** 2017-10-25

**Authors:** Pranav Kulkarni, Peter Frommolt

**Affiliations:** Bioinformatics Core Facility, CECAD Research Center, University of Cologne, Germany

## Abstract

While Next-Generation Sequencing (NGS) can now be considered an established analysis technology for research applications across the life sciences, the analysis workflows still require substantial bioinformatics expertise. Typical challenges include the appropriate selection of analytical software tools, the speedup of the overall procedure using HPC parallelization and acceleration technology, the development of automation strategies, data storage solutions and finally the development of methods for full exploitation of the analysis results across multiple experimental conditions. Recently, NGS has begun to expand into clinical environments, where it facilitates diagnostics enabling personalized therapeutic approaches, but is also accompanied by new technological, legal and ethical challenges. There are probably as many overall concepts for the analysis of the data as there are academic research institutions. Among these concepts are, for instance, complex IT architectures developed in-house, ready-to-use technologies installed on-site as well as comprehensive Everything as a Service (XaaS) solutions. In this mini-review, we summarize the key points to consider in the setup of the analysis architectures, mostly for scientific rather than diagnostic purposes, and provide an overview of the current state of the art and challenges of the field.

## 1 Introduction

**Fig. 1:**
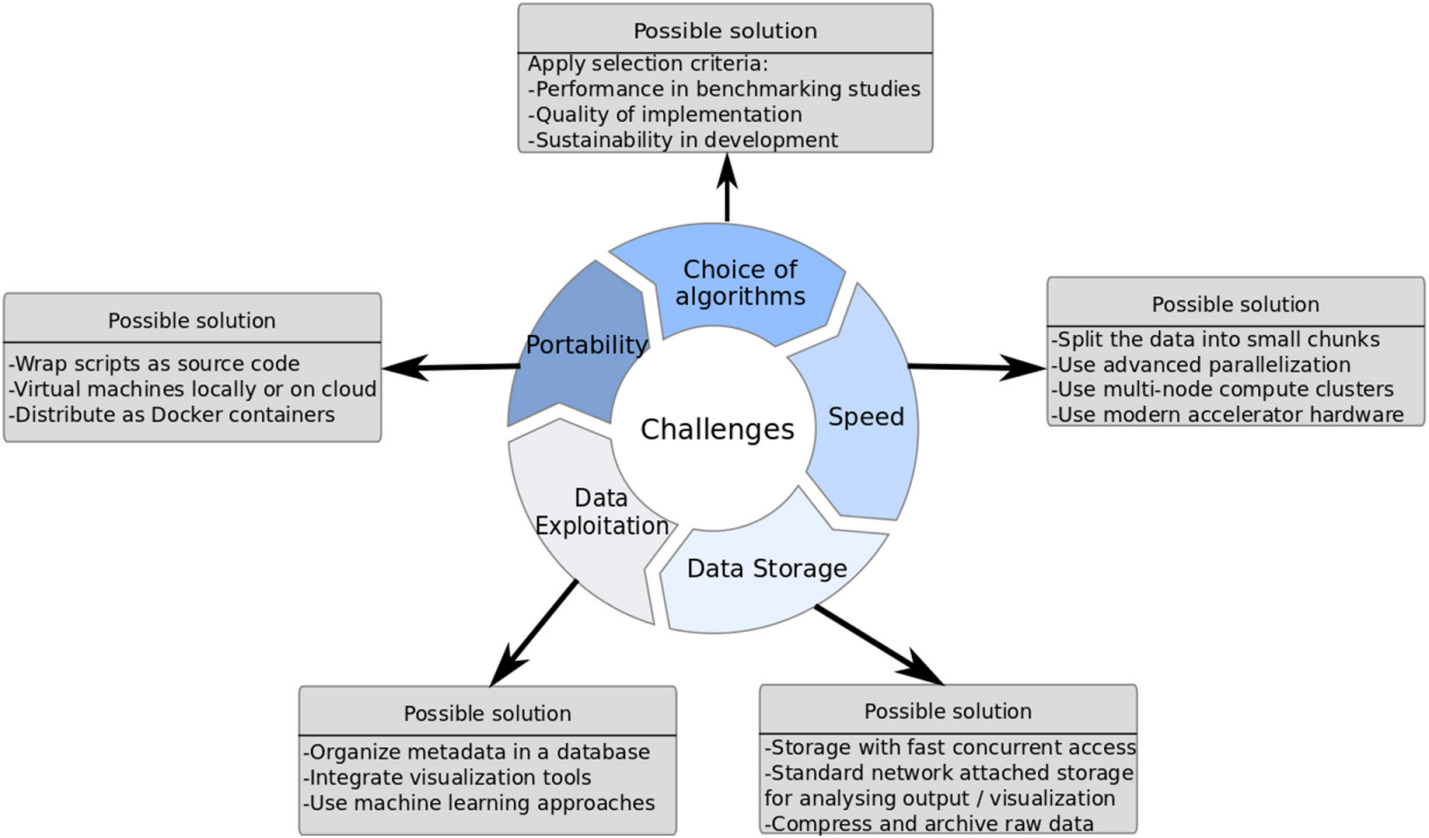
Overview of the most important challenges in the design and implementation of NGS analysis workflows and suggestions how these challenges can be addressed.

Next-Generation Sequencing (NGS) has emerged as a standard technology for multiple high-throughput molecular profiling assays. Among these are transcriptome sequencing (RNA-Seq), whole-genome and whole-exome sequencing (WGS/WXS) for instance for genome-wide association studies (GWAS), chromatin immunoprecipitation or methylated DNA immunoprecipitation followed by sequencing (ChIP-Seq or MeDIP-Seq), as well as a multitude of more specialized protocols (CLIP-Seq, ATAC-Seq, FAIRE-Seq, etc.). NGS is actually a subordinate concept for a number of comparatively new technologies. This review is focused on the analysis of data generated by the most widely used Illumina sequencing machines. Other technologies include the Sequencing by Oligonucleotide Ligation and Detection (SOLiD) method (Applied Biosystems), the 454 sequencing (Roche) and IonTorrent (ThermoFisher) machines as well as sequencers of the third generation manufactured by Oxford Nanopore and Pacific Biosciences. All these technologies are capable of generating tremendous amounts of information at base-level resolution, within relatively short time, and at low cost. These recent developments have turned the methods used in research projects into systems-wide analysis tools on organisms and diseases, which has revolutionized the paradigms followed in the life sciences in general. The appropriate selection of the right approaches to the analysis of the data is therefore a key discipline of this new era. In particular, there is a big need for clever ways to organize and process all the data within reasonable time [26] and in a sustainable and reproducible way (Fig. 1). Across most research projects as well as in many clinical environments, NGS analysis workflows share a number of steps which are the same for many use cases. Scientists around the globe have therefore established highly standardized analysis pipelines for basic NGS data processing and downstream analysis. The analysis workflows must be highly standardized, but at the same time flexible enough to also do tailored analyses and quickly adopt novel analysis methods that are developed by the scientific community.

For academic institutions, there are a number of good reasons to make investments into their own data analysis infrastructure instead of relying on commercial out-of-the-box solutions. First, academic institutions are strongly interested in having full control over the algorithms that are used for the analysis of their data. Second, the commercial solutions which do exist are either less flexible regarding their extension with additional analysis features or lack functionality and scalability to organize multiple analyses from many laboratories and very diverse research projects at a time. Third, the issue of ownership and access permissions to the data can be very important for confidential research data and patient data. This is a strong argument to not bring the data beyond the institution's firewall, e.g. by giving them to external suppliers following an Everything as a Service (XaaS) model. The requirements regarding reproducibility, validation, data security and archiving are particularly high where NGS technology is being used for diagnostic purposes. Finally, a data analysis infrastructure developed in-house provides improved flexibility in the design of the overall architecture and allows, for instance, the quick integration of novel scientific methodology into bioinformatics pipelines. On the downside, an in-house data analysis infrastructure requires significant investments regarding personnel, time and IT resources.

The optimal way to setup NGS analysis workflows highly depends on the number of samples and the applications for which data processing needs to be pipelined. The diversity of challenges in the setup of such a system are reflected by the fact that over the last years, a whole research field has emerged around new approaches to all aspects of NGS data analysis. For a small research laboratory (< 20 scientists) with a very narrow research focus, the setup may require analysis pipelines for highly specialized scientific questions at a maximum of flexibility. In contrast, an academic core facility typically needs to process data from dozens of laboratories covering multiple research fields at a sample throughput in the thousands per year. Key features of a successful analysis workflow system in such an environment are therefore resource efficiency in data handling and processing, reproducibility, and sustainability.

## 2 Data Processing

**Fig. 2:**
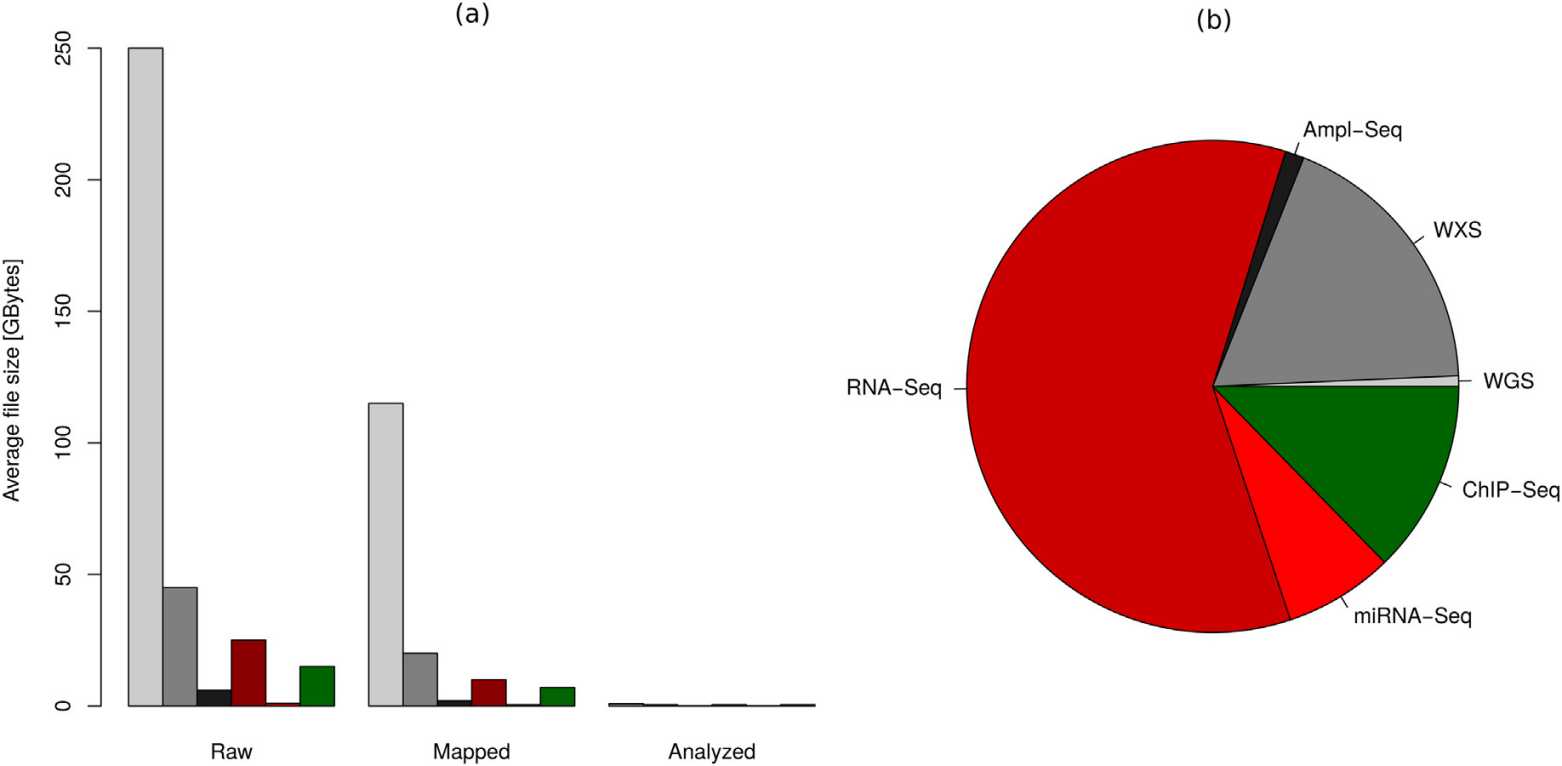
Usage of resources for large-scale analysis of Next-Generation Sequencing data in our local Core Facility: (a) Average filesystem space used for storage of NGS data at different levels of the analysis for the most important NGS applications (light grey: WGS; medium grey: WXS, dark grey: amplicon-based gene panel sequencing; dark red: RNA-Seq, light red: miRNA-Seq, green: ChIP-Seq). (b) Percentage of the analysis runs for several applications. On average, our pipelines are processing between 1000 and 1500 samples per year. (For interpretation of the references to colour in this figure legend, the reader is referred to the web version of this article.)

The unique challenge, but also the big chance in the NGS analysis field lies in the tremendous size of the data for every single sample analyzed. The raw data typically range in dozens of gigabytes per sample, depending on the application. For whole-genome sequencing, the size of the raw data can be even up to 250 GB (Fig. 2). Given sufficient computational resources, the overall workflows can be streamlined and highly accelerated by establishing centralized standard pipelines through which all samples analyzed at an academic institution are processed. State-of-the-art version control on the underlying pipeline scripts greatly improves the reproducibility of NGS-based research results in such an environment. A commonly used system for both software development and version control is *git*: the software version used for a particular analysis can, for instance, be controlled by tracking the ID of the latest git commit before the analysis has started (https://github.com).

A very basic decision is whether to build the data processing pipelines up from scratch or whether to leverage one of the existing frameworks for large-scale NGS analysis pipelining. Among the most prominent of these are the frameworks *GenePattern* [32] and *Galaxy* [12]. Both are open source platforms for complex NGS data analyses operated on cloud-based compute clusters linked to a front-end web server which enables facilitated user access. They can be used to run computational biology software in an interactive graphical user interface (GUI)-guided way and make these tools accessible also to scientists without extensive skills in programming and on a UNIX-like command line. They offer many off-the-shelf pipeline solutions for commonly used analysis tasks, which can however be modified in a flexible and interactive way. Other publicly available analysis workflow systems include QuickNGS [7], [40], Chipster [15], ExScalibur [5], and many others (Table 1). Regarding the setup of the overall architecture, the daily operation and the choice of the particular tools, especially in customized pipelines, a user of any of these systems still heavily relies on the help of experts with IT and computational biology background. Thus, the decision whether a data analysis infrastructure is build up from scratch or based on one of the aforementioned frameworks is mostly a trade-off between flexibility and the necessary investments at all levels.

**Table 1:**
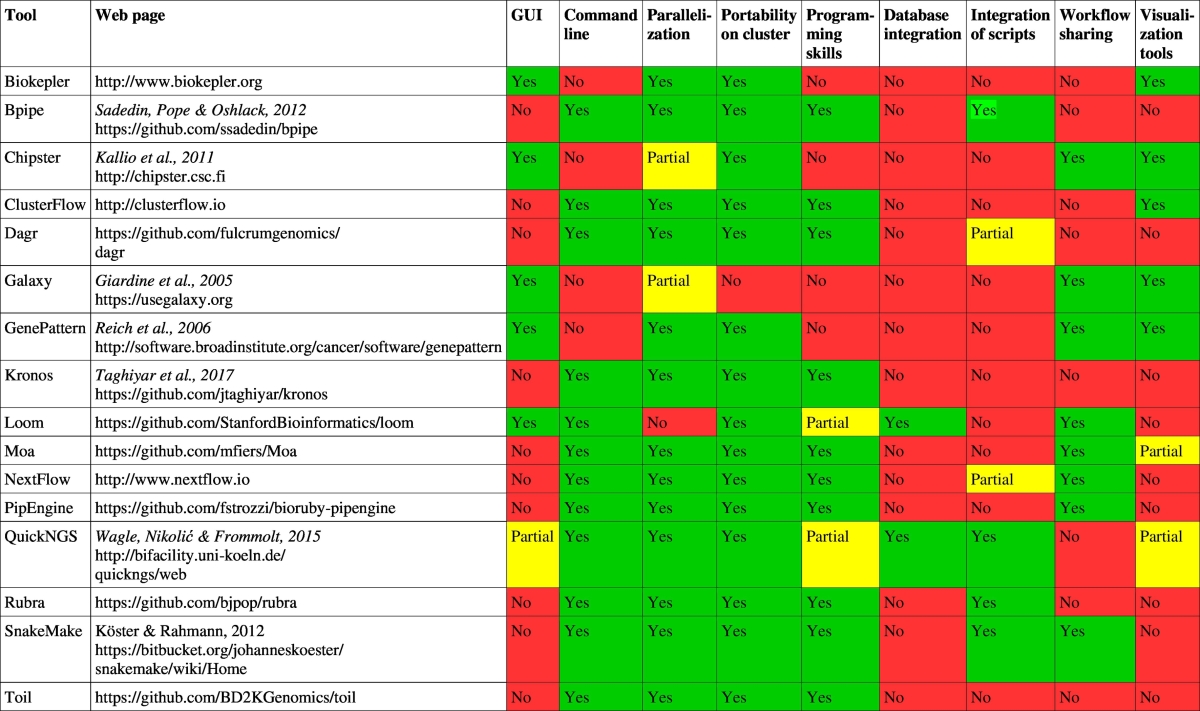
List of publicly available bioinformatics workflow systems and comparison of the features they offer.

### 2.1 Typical Steps in an NGS Analysis Pipeline

Raw data are usually provided in FastQ, an ASCII-based format containing sequence reads at a typical length of up to 100 base pairs. Alongside with the sequences, the FastQ format provides an ASCII-coded quality score for every single base. After initial data quality checks and filtering procedures, the first step in most analysis workflows is a sequence alignment to the reference genome or transcriptome of the organism of origin. For de novo sequencing of previously uncharacterized genomes, a reference-free de novo genome assembly is required. These are the most data-intensive and time-consuming step of the overall procedure and many mapping algorithms have been developed with a focus on both accuracy and speed (e.g. [10], [19], [20], [37]). The de facto standard format used for the output of these tools is the Sequence Alignment and Mapping format (SAM) and, in particular, its binary and compressed versions BAM [21] and CRAM. All subsequent analysis tasks typically build upon these formats. Among the downstream analysis steps are, for instance,

• the quantification of reads on each molecular feature of the genome in order to analyze differential gene expression or exon usage in RNA-Seq data (e.g. [4], [23], [38]),
• comparative analyses of the read coverage between a sample with a specific pulldown of epigenomic marks and an “input control” without the pulldown in ChIP-Seq, MeDIP-Seq, CLIP-Seq, FAIRE-Seq or ATAC-Seq data (e.g. [3], [44]),
• comparative analyses of the genomic sequence with that of the organism's reference sequence in order to obtain a list of potentially disease-causing mutations in whole-genome or exome sequencing data or targeted amplification/capture-based sequencing of disease gene panels [1], [6]. Further downstream steps include linkage analyses in genome-wide association studies (GWAS).

Downstream of these secondary analyses, statistical evaluation of gene clusters, networks or regulatory circuits can be provided with specialized approaches and methodology.

### 2.2 Choosing Appropriate Algorithms to Assemble a Pipeline

The most difficult and time-consuming part of the work in the setup of a new analysis pipeline is the appropriate selection of the right tools from the many existing ones. A reasonable selection criterion for a particular analysis algorithm is, for instance, its performance in published or in-house benchmarking studies (e.g. [9] [36] [43]) because the quality of the results should be quantified in a way that makes the pipeline comparable to alternative workflows. The DREAM challenges have enabled highly competitive and transparent evidence for the efficiency and accuracy of several analysis tasks, e.g. for somatic mutation calling in a tumor context [11]. The results of these competitions and benchmarking studies do therefore form a good starting point for the development of an in-house analysis strategy. It should, however, be accompanied by benchmarking studies based on data simulated in silico, but also on experimental validation of results obtained from NGS studies, e.g. by quantitative realtime PCR (qRT-PCR) or traditional low-throughput Sanger sequencing. The particular selection of parameters to the algorithms should be part of these considerations. Another very important feature of any tool that is embedded into a high-throughput analysis pipeline is the quality of its implementation and sustainability of development. In a large-scale production environment involving NGS data, it is crucial to have the workflow running fast and smoothly in almost all instances. The tools may not crash in any off-topic use cases and must use the CPU power available in an efficient way. Despite the complexity of these requirements, there are still many degrees of freedom in the actual selection and combination of analysis tools, and there can be a vast number of combinations of algorithms which are appropriate for the particular requirements.

### 2.3 Acceleration and Parallelization

In a large-scale data processing environment, the speedup of the entire analysis workflow can be an important issue, especially if the computational resources are scarce. There has been a multitude of scientific contributions aiming to speed up the overall procedure, e.g. using optimized parallelization in an HPC environment. For parallelized read mapping, the input data are typically split into small and equally-sized portions. The alignment is then carried out in parallel either by making use of array jobs [7] or by distributing the data across multiple threads using OpenMP or across multiple compute nodes using the message passing interface (MPI) [29]. This enables a significant reduction of the processing time and has been adopted in many NGS analysis pipelines (e.g. [7], [18]). However, a significant overhead caused by the time-consuming split and merge procedures are inherent in this *scatter-and-gather* approach. Furthermore, for downstream analysis, e.g. variant calling from aligned read data, the data are often split into one packet per chromosome. This usually leads to load imbalances, another well-known issue in parallel computing: the compute jobs receive different amounts of data to process and delays are caused by the waiting times until the last process has finished. This is also the case for read mapping because the time to align each read with a seed-and-extend approach can vary. In the worst case, the resources used by a computation job with unbalanced load are blocked and mostly remain in an idle state until all processes are finished.

As a consequence of these overheads and load imbalances, widely used NGS analysis pipelines do in fact show only a two- or three-fold speedup, although the increase of the resource usage compared to sequential data processing is a multiple of this. Alternative strategies in data distribution can reduce load imbalances in thus far that they scale almost linearly with the number of cores [16]. Advanced parallelization paradigms beyond MPI or OpenMP involve the Map-Reduce programming model with an open source implementation in the Hadoop framework which has been adopted, for instance, for sequence alignment [2], [30]. Given its distributed storage and processing strategy, its built-in data locality, its fault tolerance, and its programming methodology, the Hadoop platform scales better with increasing compute resources than does a classical architecture with network-attached storage [35].

The speedup achieved by parallelization is paid by an increased allocation of resources. The prevalent issues with overheads during the *scatter-and-gather* steps and with load imbalances make the usage of these resources very inefficient. To better use given resources and to avoid limitations of scalability, modern accelerator hardware architectures such as GPGPUs have been subject to research efforts in the field of bioinformatics [17], [22], [24] in order to improve the usage of existing hardware beyond parallelization. It has been demonstrated that these approaches do in fact outperform the widely used parallelization paradigms, but they have not received enough attention to set a true shift of paradigms in motion.

Finally, any efforts towards the increase of automation in all kinds of high-throughput analyses pay off in a reduced need for resources, working time as well as overall time line. The existing solutions mostly rely on file-based information supply regarding the sample names, replicates, conditions, antibodies, species in an analysis (e.g. [5], [34]). A particularly high degree of automation can be achieved if experimental meta data are modeled in appropriate databases and the reference data are organized systematically [40].

### 2.4 Workflow Portability

If a workflow has been made publicly available, it can only be adopted by other users if its installation on a different system can be completed without having to deeply dig into the source code of the software. Thus, in order to increase flexibility and usability of an NGS analysis workflow, it needs to be easy to install and execute across different platform distributions. Compatibility and dependency issues frequently occur in such cases. Recent advancements involve Docker (https://www.docker.com), which bundles an application and all its dependencies into a single container that can be transferred to other machines to facilitate seamless deployment. To increase flexibility, a workflow should be extendable in a way that users can plug-and-play their own analysis scripts into existing pipelines. A highly efficient approach can be taken by having the overall pipeline controlled by a script running in a Linux shell (e.g. the Bourne Again Shell) which provides a very powerful toolbox for file modification and analysis. Especially if parts of a pipeline have to be repeated at a later time point, it can be very efficient to organize dependencies between analysis result files by the GNU autoconf and automake concepts.

## 3 Hardware Infrastructure

Given the above considerations on parallel data processing and acceleration, an efficient NGS analysis pipeline obviously has to be built upon a non-standard hardware. The IT backbone of a platform operating on thousands of samples per year must rely on a multi-node compute cluster with exclusive access to at least part of the compute nodes. This is usually either operated by the core facility itself or by a general IT department of the host institution. In addition, cloud-based solutions for storage as well as for computational resources are on the rise following an Infrastructure as a Service (IaaS) paradigm. For instance, the Illumina BaseSpace is a solution for cloud-based data storage which can be combined with cloud computing based on the BaseSpace Apps or Amazon Web services (AWS). In order to meet their customer's security concerns, some suppliers do now offer to process all data only in nearby computing centers located at least on the same legal territory. Finally, any considerations on using a cloud-based solution or a local architecture are again a trade-off between flexibility and the amount of investments needed at all levels. One advantage of cloud-based solutions is that a significant fraction of publicly available data from large-scale consortia is immediately available on the cloud (Section 4.1).

Apart from the actual data processing, considerations on parallelization also apply to storage technologies in a file-based as well as a database environment. In NGS data processing, there are basically three kinds of storage volumes which typically play a role:

• *HPC-accessible storage*: For HPC-based NGS raw data analysis, the pure size of the data first requires that the HPC-accessible storage is of significantly high capacity, usually at least dozens of terabytes. Moreover, storage volumes used in typical NGS data processing procedures require high availability and fast concurrent access by multiple processes at a time. This is usually achieved by a storage area network based on fibre channel or iSCSI with direct access from each of the nodes in a cluster. To ensure data consistency in the presence of multiple concurrent accesses, all nodes are providing information on directory structures, file attributes and memory allocations to a meta data server which coordinates the caches and locks on the file system.
• *Long-term storage* (*high availability*): After processing has finished, large files usually have to be kept at permanent availability in order to enable scientists to use them either for downstream analysis or for visualization. At this stage, there is no need for using a file system allowing multiple concurrent accesses, but the system can rely on a standard network attached storage (NAS), e. g. by mounting it to a web server for visualization.
• *Long-term storage* (*low availability*): Once a research project has been finished or published, immediate access to the data is no more needed, but typically the data need to be saved for a further period of time. Conventional compression can reduce the size of the raw data (FastQ files) to approximately 25%. A comparatively cheap approach is to compress the raw data and archive them on a tape which can be retrieved at a later time point. This approach ensures that there is no loss of information. Given a highly standardized pipeline with state-of-the-art version control, all results can be reproduced by processing the raw data again through the original pipeline.

In order to squeeze the highest possible information out of a large resource of data, the data are ideally structured in a way which makes them accessible for high-level analyses. Many NGS analysis architectures are therefore equipped with powerful back-end databases used for storage of the processed data at a high-level structure. The approaches and frameworks to store, query and analyze genomic variation can basically be split into those based on traditional relational databases (e.g. [7], [40]) and Not-Only-SQL (NoSQL) databases. An early implementation of a scalable database system for queries and analyses of genomic variants based on NoSQL solutions was based on HBase which is employing Hadoop MapReduce for querying across a commodity cluster based on the Hadoop HDFS distributed file system [27]. Furthermore, it has been shown that the key-value data model implemented in HBase outperforms the relational model currently implemented in tranSMART [41]. NoSQL centers around the concept of distributed databases, where unstructured data may be stored across multiple processing nodes or even multiple servers. This distributed architecture allows NoSQL databases to be horizontally scalable as more hardware can be added with no decrease in performance.

## 4 Data Availability and Exploitation

### 4.1 Data Availability

Over the past decade, international large-scale consortia have employed NGS for the characterization of the human genome, its variation, dynamics, and pathology. For instance, the currently ongoing 100,000 genomes project of the British National Health Service (NHS) pursues the goal to sequence the genomes of 100,000 individuals in a medical care context with the goal to establish a population-scale genome database with clinical annotations [28]. *The Cancer Genome Atlas* (TCGA) research network has conducted multi-OMICS analyses in multiple large-scale studies on all major human cancer types with > 11,000 patients included [39]. The *Encyclopedia of DNA Elements* (*ENCODE*) and the *Roadmap Epigenomics Project* aim to establish a large-scale map of the variation and dynamics of human chromatin organization. The *4D Nucleome* initiative aims to establish a spatiotemporal map of the states and organization of the cellular nucleus in health and disease. The amounts of data obtained in these collaborative efforts are orders of magnitude higher than ever before. Beyond these large-scale consortia, an ever increasing amount of data is generated in thousands of small ongoing research projects. ArrayExpress, for instance, currently contains > 10,000 records on research projects involving RNA-Seq data. The entirety of these data reveals a highly diverse genomic landscape of the human genome and the data are hardly used in a combined way. Thus, there is a tremendous gap between the amount of data that is available and the efficiency and completeness of its exploitation to gain the best scientific benefit from it. In the next years, this gap has to be bridged by new intelligent approaches to structure the data and combine them with data from locally acquired experiments.

### 4.2 Data Security

The ability of large genomic datasets to uniquely identify individuals has been demonstrated in the past [14]. Maintaining privacy and confidentiality is thus of critical importance in the data management practice. Enforcing permission to access genomic data and storing meta data pertaining to individuals in a barcode (pseudonymization) may help protect the identity of the subjects to some extent. Local system administrators need to be informed about the location of sensitive data and potential members who are allowed to access it, such that the security of the system is constantly checked to tackle potential breaches and hacking attempts.

### 4.3 Machine Learning in Genomic Medicine

The complexity of genomic variation, dynamics and pathology cannot be modeled with a limited, human-readable number of statistical variables. Machine learning methods which have been highly effective in the analysis of large, complex data sets, are becoming increasingly important in genomic medicine. Apart from classical machine learning tasks in biology, e.g. in the field of DNA sequencing pattern recognition, similar algorithms can also operate on data generated by any OMICS assays, e. g. genomic variation based on whole-exome sequencing, gene expression data based on RNA-Seq or epigenomic data from chromatin analysis assays like ChIP-Seq. Pattern recognition algorithms on these data can be used to distinguish between different disease phenotypes and identify clinically relevant disease biomarkers.

The ultimate goal of bioinformatics architectures in medical genomics will be to adopt methodology from the field of machine learning to the analysis of processed NGS data in order to predict clinically relevant traits from molecular data. Early applications of supervised learning methods to molecular data enabled the prediction of cancer subtypes in acute leukemias based on weighted votes of informative genes [13]. Other studies employed support vector machines for the prediction of clinically relevant traits, e.g. for the classification of the primary tumor site from a metastatic specimen [31] or the prediction of post-operative distant metastasis in esophageal carcinoma [42]. State-of-the-art drug discovery pipelines could be significantly improved by deep neural networks without any knowledge of the biochemical properties of the training features [25]. In infection medicine, sequencing of bacterial isolates from patients in an intensive care unit (ICU) can lead to the discovery of multiple novel bacterial species [33], and the prediction of their pathogenicity has been carried out using machine learning trained on a wide range of species with known pathogenicity phenotype [8]. These early examples demonstrate that molecular data are generally suitable to predict clinically relevant phenotypes in yet unclassified specimens, but they all do not operate on highly structured data in sustainable databases. The infrastructures for NGS analysis and storage established in the meantime are suitable to perform data mining applications in a much more systematic, comprehensive and scalable way than previously achieved.

In the presence of massive amounts of data, machine learning techniques require theoretical and practical knowledge of the methodology as well as knowledge of the medical application field. Since new technologies for generating large genomics and proteomics data sets have emerged, the need for experts which can apply and adapt them to big data sets will strongly increase. In an ideal world, the information from which the algorithms are learning should not be limited to intramural databases, but shared between multiple centers in the same country or world-wide.

## 5 Summary and Perspectives

Despite an enormous progress of the field over the past decade, the setup of NGS data analysis workflows is still challenging, in particular, in a core facility environment where the target architecture must be able to crunch data from thousands of samples per year. Although there are now de facto standards for the basic steps in data processing, there is still a multitude of parameters to tweak, leaving behind a lot of work for the scientists working on these pipelines. As most institutions have made significant investments into the lab, IT and software infrastructure over the past 10 years, the basic procedures are now established at the world's major research institutions. Following up to the era of data acquisition, there is a strong need to also arrive in the era of data exploitation. International efforts to combine genomic data from multiple sites require strong efforts in data structuring and networking, and these topics are nowadays still in their early infancy. The next decade will bring mankind huge progress with the adoption of big data paradigms into genomic medicine to the benefit of patients.

## Conflict of Interest

The authors declare not conflict of interest.

## Transparency document

Transparency document.
